# Perinatal Hypoxia and Ischemia in Animal Models of Schizophrenia

**DOI:** 10.3389/fpsyt.2018.00106

**Published:** 2018-03-29

**Authors:** Dimitri Hefter, Hugo H. Marti, Peter Gass, Dragos Inta

**Affiliations:** ^1^RG Animal Models in Psychiatry, Department of Psychiatry and Psychotherapy, Medical Faculty Mannheim, Central Institute of Mental Health, University of Heidelberg, Heidelberg, Germany; ^2^RG Neuro- and Sensory Physiology, Institute of Physiology and Pathophysiology, University of Heidelberg, Heidelberg, Germany; ^3^RG Neurovascular Research, Institute of Physiology and Pathophysiology, University of Heidelberg, Heidelberg, Germany; ^4^Department of Psychiatry, University of Basel, Basel, Switzerland

**Keywords:** perinatal, hypoxia, schizophrenia, ischemia, animal models, neurodevelopment, vannucci

## Abstract

Intrauterine or perinatal complications constitute a major risk for psychiatric diseases. Infants who suffered from hypoxia–ischemia (HI) are at twofold risk to develop schizophrenia in later life. Several animal models attempt to reproduce these complications to study the yet unknown steps between an insult in early life and outbreak of the disease decades later. However, it is very challenging to find the right type and severity of insult leading to a disease-like phenotype in the animal, but not causing necrosis and focal neurological deficits. By contrast, too mild, repetitive insults may even be protective *via* conditioning effects. Thus, it is not surprising that animal models of hypoxia lead to mixed results. To achieve clinically translatable findings, better protocols are urgently needed. Therefore, we compare widely used models of hypoxia and HI and propose future directions for the field.

## Introduction

Schizophrenia (Sz) is a severe psychiatric disease affecting approximately 1% of world population. Sz patients suffer from positive symptoms such as delusions and hallucinations, negative symptoms including affective flattening and social withdrawal as well as cognitive deficits (1). While the disease usually manifests in late adolescence or young adulthood, it is commonly assumed that insults lay its pathophysiological foundation already early in development (2). Intrauterine or perinatal hypoxia–ischemia (HI) is a well-known epidemiological risk factor for Sz, increasing the hazard ratio twofold (3). Young brains react highly plastic to transient hypoxic insults below apoptotic threshold (4). Several hundred genes are known to be upregulated in response to hypoxia and trophic factors such as BDNF are secreted. In genetically predisposed individuals, this neuroprotective response may be compromised, rendering their brain more vulnerable to HI insults (5, 6). While a healthy infant may fully recover from mild transient HI, an insult of similar severity in an infant with a compromised neuroprotective response may cause long-lasting deficits. Indeed, many Sz-associated genes are involved in HI responses (7), and fetuses at high risk for psychosis appear to be more vulnerable to hypoxia (8). If only most vulnerable cell populations are affected and the functional alterations are subtle, they may result in a subclinical phenotype with an intrinsically higher vulnerability to environmental and pharmacological stress. Exposure to such stress in adult life ultimately leads to clinical manifestation (9). However, the steps between an early-life insult and the outbreak of the disease decades later are unknown. To advance beyond hypotheses and dissect the underlying mechanisms, proper animal models of perinatal HI are needed. They are required to reliably resemble the human condition as closely as possible including neurophysiological alterations similar to those in patients and a psychosis-like phenotype. In this article, we discuss the models currently employed in the field and point out possible future directions.

## HI Models for Psychiatric Research

### Important Considerations for the Design of the Study

The attempts to achieve clinically translatable results are hampered by the challenge to titrate the desired insult severity according to the vulnerability of the studied system. Depending on the condition to be modeled, deficits ranging from subtle functional alterations to wide-spread necrosis may be pursued. The latter can be relatively easily evaluated by common histological means and neurological signs. The detection of former, however, as needed in Sz models, requires a careful choice of specific readout parameters.

#### Parameters Affecting Insult Severity

In HI models of Sz, the insult needs to be severe enough to elicit chronic measurable functional deficits, without causing wide-spread necrosis and focal neurological deficits. On the other hand, repetitive mild HI may lead to conditioning effects, which increase the resilience to further stress (10). The severity of the HI insult depends on the following parameters: oxygen (O_2_) concentration and composition of the atmosphere, duration of hypoxia/anoxia, presence or absence of ischemia, and presence or absence of an adaptation period. While anoxia and severe hypoxia below 4% O_2_ can be survived for only several minutes, milder hypoxia between 6 and 12% O_2_ can be tolerated for considerably longer periods of time (as up to days or weeks), especially if the insult is preceded by an adaptation period (11). Interestingly, there is a difference between anoxia with 100% N_2_ vs. anoxia with 100% CO_2_ in the atmosphere, the latter causing a stronger release of free radicals as shown in mice on their first postnatal day (P1) (12). If ischemia is induced additionally to hypoxia, the tolerance time may be dramatically shortened; thus, the insult duration should be adjusted.

#### Parameters Affecting System Vulnerability

Fundamental differences between species need to be carefully considered. Although mammals including pigs, sheep, and primates may reassemble human condition more closely, rodents are the most common model organisms in HI research. But even in ontogenetically related species such as mice and rats, the same kind of insult can lead to very different peripheral and CNS responses (13, 14). As preterm or perinatal insults are considered as risk factors of Sz, the insult needs to be administered at the according developmental age. During the first two postnatal weeks, the CNS in rodents is highly dynamic and differently reacts to environmental insults (15). Methods to translate the developmental age across different species have been developed (16). According to this model, the CNS of a P7–P8 mouse pup developmentally corresponds to a preterm infant in the third trimester. This period is crucial for dendritic outgrowth, synapse formation, maturation of neurons, and interneurons particularly (17). Furthermore, synchronized network function in form of rapidly evolving oscillations emerges at this age (17). They are accompanied by fundamental changes in synaptic transmission such as the switch of NMDA receptor (NMDAR) subunits GluN2B to GluN2A (18) and the transition of the excitatory to inhibitory action of GABA (17). Thus, it is plausible that such fundamental features may dramatically influence neuronal vulnerability. Differences in the development of brain areas and cell types also need to be taken into account (16). Furthermore, strain (19) and sex (20, 21) differences need to be considered. The vulnerability to HI highly differs between brain areas and cell types (22). Thus, hippocampal CA1 pyramidal cells belong to the most susceptible neurons, while the granule cells in the dentate gyrus are very resistant to HI insults (23). Parvalbumin-expressing fast-spiking interneurons are also regarded as highly vulnerable to HI damage due to their high energy demand (24), although the available data are conflicting, possibly due to the heterogeneity of these neurons (25, 26). Last but not least, peripheral factors such as body temperature, respiratory reserve, cardiovascular regulatory mechanisms, blood composition, immune and hormonal response, and environmental cofactors affect the vulnerability of the organism and should be controlled for as thoroughly as possible as they may fundamentally influence the outcome (27–30).

#### Important Readout Parameters for Sz Research

Proper readout parameters for Sz-like alterations on anatomical, functional, and behavioral level need to be carefully selected. On the behavioral level, increased locomotion, stereotypical movements, hyperirritability, deficits in social interaction, various cognitive deficits, and sensorimotor gating deficits following perinatal HI are implied to represent a psychosis-like phenotype in rodents analogous to clinical symptoms of Sz (31). Similar to Sz patients, rodents exposed to HI exhibit increased sensitivity to stimulants such as amphetamines, while antipsychotics ameliorate many of their deficits (31). However, behavioral studies in rodents are naturally limited as they will never fully represent the wide range of human symptoms. Furthermore, the specificity of a behavioral phenotype needs to be critically scrutinized since the partial overlap of phenotypes in models of different psychiatric diseases cannot be neglected (32). To improve construct validity of animal models, it is therefore crucial to underpin a behavioral phenotype by pathophysiological and anatomical traits that characterize the specific human disorder. Importantly, HI models display several such traits of Sz. Thus, perinatal HI leads to cortical thinning, ventriculomegaly, and reduced hippocampal volume (33), all findings present in Sz patients (34–36). Furthermore, HI induces cell loss in hippocampal CA1 and CA3. Reduced hippocampal volume correlates with the severity of positive symptoms and required antipsychotics dosage (37). These anatomical changes are accompanied by functional deficits including disrupted communication between large cortical networks (38, 39), altered cortical oscillations (40, 41), and sensorimotor gating deficits (42). Novel electrophysiological and imaging techniques confirmed the presence of these endophenotypes in rodent models (42–44). Loss and/or malfunction of GABA-ergic interneurons, particularly parvalbumin-expressing fast-spiking interneurons, is considered a hallmark of Sz on the cellular level (45, 46) and provides a simple and important histological readout in animal models. On the molecular level, NMDAR hypofunction and hyperdopaminergia are considered to underlie Sz pathophysiology (47, 48). Expression, structure, and function of glutamate and dopamine receptors are therefore important objects of study with electrophysiological and molecular biological methods. HI indeed was found to induce changes in glutamatergic and dopaminergic transmission (49). In summary, a multiple-scale approach employing a combination of different techniques should be used to detect Sz-characteristic changes in cellular, network, and whole-brain functions.

### Common Experimental Paradigms

The common animal models can be divided into two major groups: pure hypoxia and combined HI models. The first group can be further subdivided into chronic intermittent, chronic continuous and acute hypoxia (AH)/anoxia models. Hypoxia is usually achieved by placing the animal into a sealed chamber where the atmosphere content can be precisely regulated with a gas delivery system. In this article, we will focus on four commonly used types of models in rodents and will omit discussion of rarer models such as asphyxia or submersion as well as models in other species.

#### Chronic-Intermittent Hypoxia (CIH)

In CIH models, the animals are intermittently exposed to hypoxia of usually 6–10% O_2_ on several consecutive days. This relatively mild insult usually leads to subtle functional and behavioral alterations without presence of necrotic cell death. A paradigm employing a 6–18 h hypoxia (11% O_2_)—normoxia cycle in P4–P8 rats led to prepulse inhibition deficits (a measure of sensorimotor gating deficient in Sz patients) and locomotor deficits (regarded as a correlate of positive symptoms) (50). This syndrome was reversed by clozapine (51). These deficits correlated with altered expression of the NMDAR and various synaptic proteins (26, 50). In another study employing a similar paradigm, CIH led to impairments in hippocampal long-term plasticity that was rescued by BDNF administration (26). A CIH model with 10% O_2_, 5% CO_2_, and 85% N_2_ in P7–P11 rats led to long-lasting hyperlocomotion, working memory impairments, and circadian rhythm abnormalities that were accompanied by upregulation of striatal D1 receptors (52). Altered dopaminergic function in animal models corresponds to findings in human neonates and may lay a pathophysiological foundation for Sz in later life (53). In contrast to these results, a study conducted by our group did not find protracted behavioral abnormalities after CIH with 10% O_2_ 6 h/day in P3–P7 mice (54). This lack of deficits may be due to compensatory mechanisms such as induction of neurogenesis (55, 56), expression of neurotrophic factors such as BNDF (57) and conditioning effects (58). While the previous studies utilized relatively mild hypoxia, it is also feasible to administer brief periods of anoxia in a chronic-intermittent manner. One such paradigm caused specific cognitive and emotional deficits including reduced attention and increase in anxiety in absence of detectable structural damage (59).

#### Chronic Continuous Hypoxia (CCH)

Chronic continuous hypoxia models usually employ hypoxia in the range of 6–12% O_2_ which is administered continuously on several consecutive days. Various behavioral phenotypes have been reported in CCH models including hyperactivity, increased aggression, and altered adult sexual behavior (60, 61). 10% O_2_ CCH from P1 to P20 leads to reduced CA1 cell counts (60). 9.5% O_2_ CCH in P3–P11 rats causes ventriculomegaly and cortical thinning (62), findings also observed in human Sz patients (35). Our group, however, was not able to replicate these results in mice, possibly due to adaptive and conditioning effects described above (55) or higher hypoxia tolerance of the species (54). Moreover, a study reported an increased numbers of cortical neurons following chronic hypoxia of 9.5% O_2_ (63), indicating reduced cell death or increased neurogenesis following the HI insult (64).

#### Acute Hypoxia

In these models, severe hypoxia or anoxia is administered. Depending on the oxygen content and insult duration, it is possible to model different conditions. AH of 3–4% O_2_ for 15–20′ as well as 10′ of anoxia reliably induce seizures in rat pups (65, 66). Further effects are reduced CA1 cell density and behavioral abnormalities in early adulthood including hyperactivity, susceptibility to stress, disturbed motor coordination, and enhanced startle response (60, 67–69). However, some studies indicate that already a caesarian section alone may suffice to cause alterations in the dopamine system and vulnerability to stress (70). P1–P2 rat pups survived anoxia of up to 25′ without morphological changes, but developed hyperactivity and performed worse in passive avoidance tasks in later life (71). These deficits correlated to persistent abnormalities in monoamine systems and hyperresponsiveness to amphetamines and stress in later life (72, 73). They were rescued by blocking NMDAR with MK-801 before anoxia (71). By contrast, a short anoxia of 5′ was protective and induced neurogenesis in P1 rats (74). Birth anoxia furthermore induced persisting spatial memory deficits and altered expression of the calcium binding protein parvalbumin in interneurons of the hippocampus (28). Intriguingly, P8 in rats, neurodevelopmentally corresponding to approximately pre-birth human age, was found to be a highly vulnerable period (22). Few hours of 7–8% O_2_ at this age leads to reduced brain growth, cortical thinning (22), alterations of the cholinergic, serotonergic, and dopaminergic systems, and disturbed sleep (75, 76).

#### The Rice-Vannucci Model of HI (RVM)

In most experimental paradigms, HI conditions are achieved using the RVM, first published in 1981 (77). In this model, unilateral ligation of one common carotid artery is followed by mild hypoxia in a sealed chamber. Due to sufficient collateralization, ligation itself does not lead to tissue damage. Only upon combination of ischemia and hypoxia, the oxygen and glucose supply to the ipsilateral hemisphere is compromised to a degree enough to evoke functional and structural deficits (78). The severity of injury depends on the duration and severity of hypoxia (79). A commonly used paradigm of 8% O_2_ for up to 3 h leads to combined apoptotic and necrotic cell death in parts of the neocortex, hippocampus, striatum, and the white matter (80, 81). Importantly, the contralateral side appears unimpaired and can serve as internal control (82). The RVM gives rise to various behavioral phenotypes including motor deficits (81), sensory processing abnormalities (83), impaired spatial learning and memory (81, 84), and reduced attention (83–85). Interestingly, these deficits appear to be hemisphere specific and sex dependent as lesions of the right hemisphere were found to cause larger impairments in working memory tasks (86), while females with left occlusion presented greater spatial memory impairment and histological damage than males with left occlusion or females with right occlusion (21).

## Discussion

**Figure 1:**
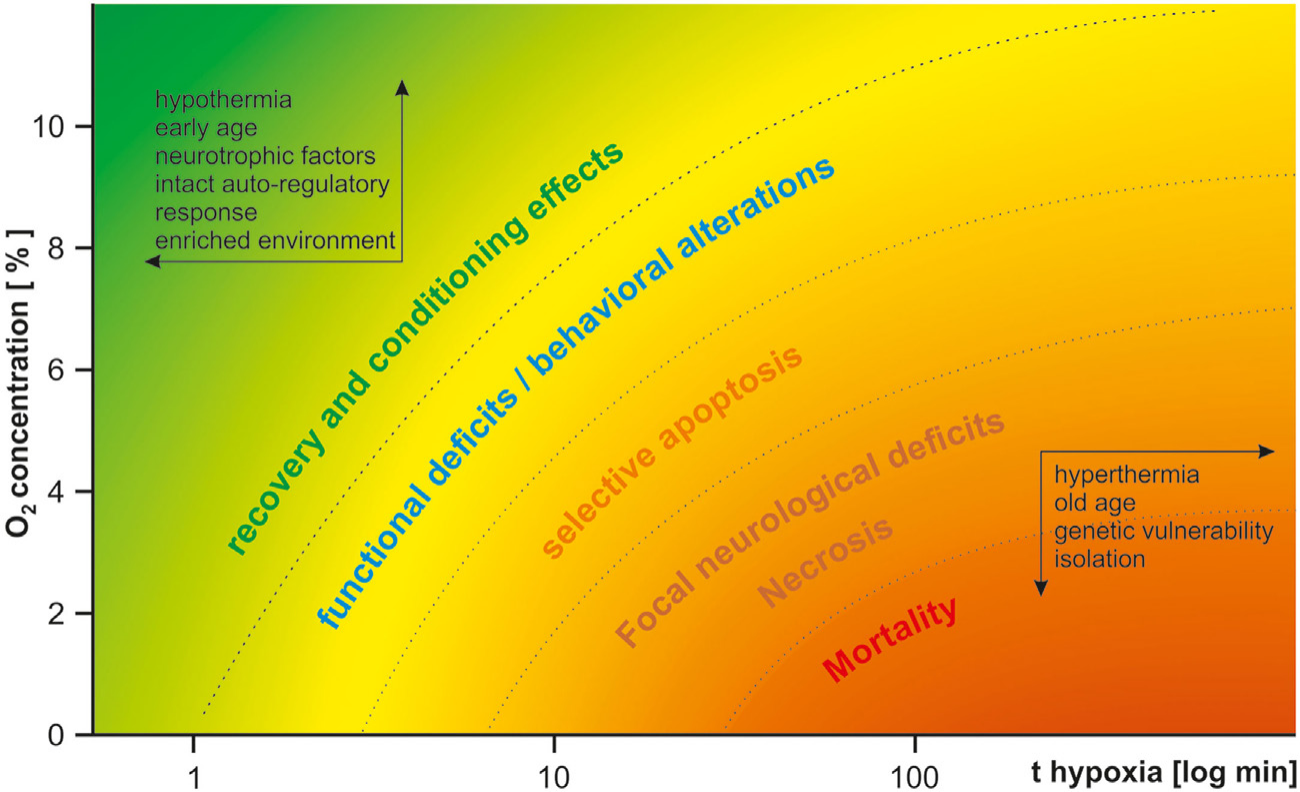
Qualitative effects of hypoxia in dependence of severity and duration of the insult. The insult duration is drawn logarithmically on the *x*-axis. The oxygen concentration is drawn on the *y*-axis. Depending on both parameters, the insult either lacks long-lasting effects or is even protective (upper-left corner, green), or leads to functional and structural deficits (from yellow to orange) and mortality (bottom-right corner, red). Protective factors are listed within the arrows in the upper-left corner; harmful factors are listed within the arrows in the bottom-right corner. For psychiatric research, a mild type of insult in the yellow range needs to be carefully selected.

Perinatal HI brain damage can induce a broad variety of psychiatric and neurological symptoms, depending on the kind and severity of the insult and vulnerability of the system studied. Figure 1 gives a qualitative overview of HI effects depending on duration and severity of hypoxia. In light of the multitude of co-variables, it is hardly surprising that the same model may yield contradicting results. This variance may be reduced by thorough standardization of procedures. To address basic questions on the molecular, cellular, and local network levels and for studies of metabolism, *in vitro* models of HI may be more suitable because of better control or even exclusion of many of the environmental variables listed above (87, 88). As the local circuitry and cytoarchitecture are preserved in acute brain slices, they provide a tool to study the effects of HI within a frame of up to 12 h, while organotypic slice cultures enable the study of chronic changes of up to 1 month (88–90). Despite all difficulties, perinatal HI models are valuable for Sz research, given a suitable model is employed to optimize specificity and construct validity, as many features of human patients can be mimicked. Therefore, instead of the question “whether,” the question “which—HI model(s) is/are useful in Sz research,” is more appropriate. Severe HI models are more widely employed in neurology while mild chronic continuous or intermittent hypoxia models are more established in psychiatry because of the desired lack of cell death in the latter. However, mild models suffer from higher variance in results and reduced reproducibility. We suppose that such mild insults may be on the watershed between causing subtle functional deficits and evoking compensatory protective effects *via* conditioning, gene expression, and periphery counter-regulation mechanisms. Especially mild CIH models may succumb to these conditioning effects. HI alters expression of hundreds of genes, including BDNF (57) and amyloid precursor protein both in rodents and humans (91), which is supposedly part of an acute compensatory protective response, but may lead to dementia in later life (92). The neuroprotective effects of repetitive mild HI are well described in humans and animal studies (93). Transient HI induces tolerance to further HI insults and is subject of investigations as a possible therapeutical tool in neurological and cardiological clinical trials (94, 95). The translational potency of AH models in P0–P1 rodents is questionable, as this age neurodevelopmentally corresponds to the early second trimester in humans. On the other hand, mild CCH from P1 to P20 also hardly reproduces any human pathophysiological condition, translating to a human age of second trimester to late childhood. Therefore, we advocate the use a single HI insult in close resemblance to human condition in its core features such as duration and severity. As highlighted previously, it is furthermore crucial to use rodents at a proper developmental stage (P7–P8, corresponding to preterm infants or the third trimester or P9–P10 rodents, corresponding to the perinatal period) (16). While usually employed in neurological research, the RVM of HI may be successfully adapted for psychiatric models by titrating the insult (by reduction of hypoxia duration or elevation of the O_2_ concentration) in a way that focal necrosis does not occur. It may prove more reliable than pure hypoxia models as chronic adaptive effects could be circumvented. Compromised perfusion of the brain combined with sub-necrotic hypoxia of several minutes to few hours appears to quite well reassemble human obstetric complications. Furthermore, the multifactorial etiology of the disease needs to be appreciated. Thus, perinatal insults constitute only one of multiple risk factors of Sz and may lead to its manifestation only in combination with hereditary vulnerability and further environmental stressors in later life. Therefore, development of two-hit or three-hit models should be sought in addition to the HI insult, including a pharmacologically or genetically induced risk constellation and environmental factors such as social stress or isolation.

## Author Contributions

DH, HM, PG, and DI contributed conception and design of the study; DH wrote the first and the revised drafts of the manuscript and drew the figure. All the authors significantly contributed to manuscript revision, provided important intellectual content, and read and approved the submitted version.

## Conflict of Interest Statement

The authors declare that the research was conducted in the absence of any commercial or financial relationships that could be construed as a potential conflict of interest.
